# A 3D Hand Attitude Estimation Method for Fixed Hand Posture Based on Dual-View RGB Images

**DOI:** 10.3390/s22218410

**Published:** 2022-11-01

**Authors:** Peng Ji, Xianjian Wang, Fengying Ma, Jinxiang Feng, Chenglong Li

**Affiliations:** School of Information and Automation Engineering, Qilu University of Technology (Shandong Academy of Sciences), Jinan 250353, China

**Keywords:** three-dimensional hand attitude estimation, dual-view RGB image, ensemble learning, convolutional neural network

## Abstract

This work provides a 3D hand attitude estimation approach for fixed hand posture based on a CNN and LightGBM for dual-view RGB images to facilitate the application of hand posture teleoperation. First, using dual-view cameras and an IMU sensor, we provide a simple method for building 3D hand posture datasets. This method can quickly acquire dual-view 2D hand image sets and automatically append the appropriate three-axis attitude angle labels. Then, combining ensemble learning, which has strong regression fitting capabilities, with deep learning, which has excellent automatic feature extraction capabilities, we present an integrated hand attitude CNN regression model. This model uses a Bayesian optimization based LightGBM in the ensemble learning algorithm to produce 3D hand attitude regression and two CNNs to extract dual-view hand image features. Finally, a mapping from dual-view 2D images to 3D hand attitude angles is established using a training approach for feature integration, and a comparative experiment is run on the test set. The results of the experiments demonstrate that the suggested method may successfully solve the hand self-occlusion issue and accomplish 3D hand attitude estimation using only two normal RGB cameras.

## 1. Introduction

Hand posture has a wide range of applications in human–computer interaction, augmented reality, virtual reality, and gesture recognition [1,2,3]. With the development of new human–computer interaction methods towards a more natural and convenient trend, hand-posture-based interaction has a very important research significance and prospects in the fields of game entertainment, medical treatment, smart home, military field, and so on [4,5]. The key to using hand-posture-based human–computer interaction is accurate gesture estimation. Hand pose estimation and hand attitude estimation are two categories of gesture estimation. The purpose of hand pose estimation is to precisely pinpoint the locations of the hand node points to deduce the relevant hand posture from the position data. It is frequently used in situations requiring high degrees of freedom for human hand node points, including 3D somatosensory games, 3D modeling, and other circumstances. The goal of hand attitude estimation is to predict the 3D attitude angle of a fixed hand posture in 3D space, it is more suitable for applications where the degree of freedom of the target object is low.

There are many kinds of research works on hand pose estimation, which can be divided into wearable-sensor-device-based methods and computer-vision-based methods [6,7]. Wearable-sensor-based hand pose estimation methods require the user to wear sensor-equipped data gloves or other external assistive devices to directly obtain the position coordinates of hand nodes with the help of sensor components and then give the corresponding hand pose estimation based on the position coordinates of the nodes. This method is less susceptible to natural environmental factors such as illumination and background and has good robustness and stability. However, assistive devices are generally expensive and restrict movements, which affects the operation of other handheld devices, such as robot controllers, weapons, etc.

With the technological progress of visual equipment as well as big data and artificial intelligence (AI) algorithms, it is possible to acquire large-scale image data, which has made more and more researchers begin to study vision-based and AI-based hand pose estimation [8]. Such methods get rid of the constraints of wearable devices on hands and the mismatch between device size and users’ hands and are more flexible to use. According to the different spatial dimensions of the output results, it can be divided into 3D hand pose estimation and 2D hand pose estimation. The 3D hand pose estimation methods are mostly designed for depth images acquired by depth cameras, which can directly reflect the geometry of the visible surface of an object as the depth image itself carries the depth information of the target object, which largely facilitates the study of pose estimation. However, most commercially available depth cameras are based on structured light technology, binocular stereo vision technology, and time-of-flight methods, which are extremely sensitive to environmental factors such as lighting and are suitable for special scenarios such as being used indoors. These methods require certain specifications for depth cameras and depth images and are less portable. Compared to depth images, RGB images are more applicable, can be freely acquired and applied on mobile phones, computers, and other electronic devices, are highly portable, have low environmental requirements, and are inexpensive. However, due to the lack of depth information in RGB images and the difficulty of accurate 3D data annotation (which is a major challenge for 3D hand pose estimation), most studies only implement 2D pose estimation on RGB images. In addition, the high degree of freedom of the human hand and the severe self-occlusion are still difficult problems to overcome in hand pose estimation. Therefore, the study of 3D hand pose estimation only from 2D images is of great research significance and challenging.

Furthermore, we discovered through analysis that the degree of freedom of the controlled object was not high in many hand-posture-based human–computer interactive teleoperation applications, such as vehicle-mounted pan–tilt–zoom (PTZ) camera systems [9], military reconnaissance robot systems, and a variety of robotic arm control systems. Remote operation can be achieved by using a fixed hand posture without using very complex and diverse hand postures. Inspired by this, we offer a method for a fixed hand posture that can directly regress the three-axis attitude of the fixed hand posture rather than anticipating the nodal location of all hand joints. By doing this, we may reduce the complexity of the hand model for hand pose estimation while still achieving comparable outcomes in various hand-posture-based applications.

In this research, we present a CNN- and LightGBM-based 3D hand attitude estimation method using dual-view RGB images for a certain fixed hand posture based on the aforementioned issue and the existing state of affairs. This technique uses two monocular cameras and an IMU sensor to automatically annotate the three-axis attitude labels on a huge dual-view image dataset. A deep ensemble model for estimating the 3D hand attitude is trained using the annotated dataset. To achieve this, we first utilize a CNN to automatically extract the hand image’s deep feature data. We then decide to utilize an ensemble learning approach to fit the hand attitude regression to the feature data since utilizing one model for decision-making tends to suffer from a certain level of randomness and uncertainty, that is, a certain amount of variance and bias in one model. The method is empirically tested, and the findings demonstrate that the trained model has good generalizability, is more accurate in tests than other competing algorithms, and produces good real-time results.

The main contributions of this paper can be summarized as follows:

1. A large-scale multiview hand posture dataset construction and automatic annotation is proposed, which has low hardware equipment requirements and is easy to operate.
2. A novel ConvLGBM PoseNet based on a CNN and LightGBM is proposed to predict the three-axis attitude angles of a fixed hand posture, which only needs two monocular cameras and can effectively weaken the influence of hand self-occlusion, realizing 3D hand attitude estimation on 2D images.
3. A novel Bayesian optimization for a hand attitude regressor is proposed, which significantly improves the prediction accuracy of the model with the optimal hyperparameters.

The structure of this paper is organized as follows. We first briefly describe the related works in Section 2. In Section 3 and Section 4, we introduce our method. Our algorithm is evaluated in Section 5, and the conclusion is given in Section 6.

## 2. Related Works

Computer-vision-based hand pose estimation methods can be divided into 2D and 3D according to the spatial dimension of the output data, and either type of hand pose estimation can be divided into model-driven and data-driven methods. A model-driven method is to construct a model based on the prior knowledge of hand structure to compare the difference between the generated hand image and the actual image, establish the distance measurement loss function between the actual hand and the player model, and adopt the optimization idea to minimize the loss. De La Gorce et al. [10] proposed a deformed hand triangulation surface using a model-driven method based on the RGB image alone to achieve 3D hand pose estimation. However, the optimization process based on a model-driven method had a large amount of calculation, and its accuracy depended on the artificial similarity function.

On the contrary, a data-driven method does not require complex model calibration, but it requires a large sample of training data from which the mapping relationship between hand images and hand configurations is automatically learned. This type of method can be further divided into detection-based and regression-based methods. The detection-based method is to generate a heat map to get the final predicted node joints [11,12], while the regression-based method is to directly regress the node position of all hand joints. A deep network was proposed in [13], which used a segmentation network to locate the hand, and output the scoring map of each joint point by PoseNet. In [14], an Openpose model was first used to estimate the 2D position of the hand joints, and then a 3D model of the hand was fitted to the estimated 2D joint positions using nonlinear least squares to recover the 3D hand pose. Most of the existing regression-based hand pose estimation regress the position coordinates of the hand joints, and there have been no studies that directly regress the 3D pose angles.

To solve the problem that the self-occlusion of the hand and the high degree of freedom affect the prediction accuracy of the model, in [15], the author proposed an improved algorithm for hand key node detection called multiview guidance and it achieved a high accuracy of detection. Deying Kong et al. [16] proposed a rotation-invariant mixed graphical model network, which overcame the self-occlusion problem of the hand to a certain extent. Although these methods can effectively alleviate the self-occlusion problem of gestures, they also increase the complexity of the model. Aiming at the above problems, we used dual-view images to improve the prediction performance of hand attitude estimation without increasing the complexity of the model; this paper trained two independent hand attitude estimation models for two single-view images, respectively, to weaken the problem of hand self-occlusion by reducing the variance of the model.

In the data-driven pose estimation method, a high-quality data set is one of the important factors that determine the performance of the model. In addition, using ensemble learning for model fusion can also improve the accuracy of hand pose estimation. Most of the existing pose estimation methods are designed based on a single machine learning model architecture, but the training of the model has a certain randomness, and it is easy to produce the phenomenon of overfitting or underfitting. In recent years, ensemble learning algorithms have achieved good results in various classification and regression tasks. They fuse multiple models into a learning model with a stronger robustness and generalization performance according to a certain strategy. Ensemble learning has two joint fusion strategies: bagging [17], which improves model performance by reducing variance, and boosting [18], which focuses on reducing bias. The representative algorithm of bagging is the random forest [19], Adaboost is a representative boosting algorithm, and another representative algorithm is GBDT. In 2016, Tianqi Chen proposed XGBoost [20] based on GBDT, and XGBoost added a regularization term to the loss function to prevent overfitting, it presorted the values of features before finding the best split nodal point and supported column sampling. In 2017, Microsoft Labs made a series of optimizations to the XGBoost algorithm and proposed LightGBM [21]. This ensemble learning algorithm has become one of the most dominant algorithms in the Kaggle competition due to its excellent performance and is widely used in real-world industries.

To our knowledge, there has been little research using ensemble learning ideas for hand pose estimation. Among them, a five-layer integrated neural network based on the layered idea was proposed in [22], which decomposed the hand pose estimation task into five single-finger pose estimation subtasks and fused the subtask estimation results to estimate the full 3D hand pose. A deep ConvNet architecture called a regionally integrated network was proposed in [23] for end-to-end optimization and inference to regress 3D hand joint coordinates. These studies inspired us to use ensemble learning to improve the performance of hand pose estimation.

In addition to the weak learners commonly used in ensemble learning, some studies also integrate strong learners, such as neural networks, the integration strategy of which can be divided into two kinds. One is end-to-end training, which uses the strategy of ensemble learning to fuse multiple CNN-based learners directly. Refs. [24,25,26] used a CNN as the base learner using the integration strategy of Adaboost to train the integration model in different application contexts. Ji Peng et al. [27] proposed a CNN-SVM integrated classifier on a gesture recognition task, using SVM to replace the fully connected layer in the CNN and then using the bagging algorithm to integrate the CNN. Another option is extracting image features with a CNN and then integrating the features using an ensemble learning algorithm. A new image classification algorithm based on the combination of CNN and XGBoost was proposed in [28]. The same study in [29,30,31] used a CNN to extract image features and then used XGBoost for integration. In summary, previous research has established that the fusion of strong learners by using ensemble learning can further improve model performance.

## 3. Framework Overview

In some applications of visual-based hand posture teleoperation, such as a pan–tilt–zoom (PTZ) with two-degree-of-freedom, the controlled object does not require much freedom, so it can be controlled with a simple fixed hand posture. We propose a 3D hand attitude estimation method based on deep learning and ensemble learning using dual-view RGB images for a certain fixed hand posture. This method constructs an integrated network structure (ConvLGBM PoseNet) for hand attitude estimation based on a CNN. It can directly predict the three-axis attitude angles of the hand from the RGB image to realize the hand posture teleoperation of the controlled device. We used a CNN as the basic framework of this hand attitude estimation model. First, we extracted the depth features of the hand image and then used the ensemble learning algorithm as the top-level hand attitude regressor to replace the fully connected layer in the CNN. The structure combined the automatic feature extraction capability of CNNs and the advantage of ensemble learning models to overcome random interference to establish a strong mapping between the hand image set and the hand attitude angle set. It skillfully adopted the strategy of ensemble learning to improve the performance of the CNN. Compared with a single CNN, it could overcome random interference.

**Figure 1:**
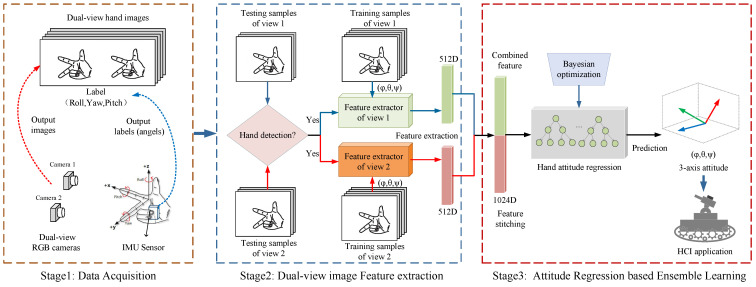
Overview of 3D hand attitude estimation method based on ConvLGBM PoseNet.

Figure 1 illustrates the overall structure of this paper’s hand attitude estimation method. The dual-view RGB image used in this method was collected by a dual-view camera composed of two ordinary monocular cameras that can output RGB images. There was no strict requirement for the position of the two cameras concerning each other, only that the hand image could be captured simultaneously. Different from ordinary binocular cameras, this type of dual-view camera does not require calibration and line justification. Using this dual-view camera, we could capture 2D images with hand posture in two different views, which could effectively reduce the hand self-occlusion phenomenon. The model trained with this dual-view hand image could effectively reduce the prediction error caused by hand self-occlusion.

The structure mainly included three parts: data acquisition, feature extraction based on a CNN, and attitude regression based on ensemble learning. The first stage was the process of acquiring data and building our dataset. We designed a dataset acquisition scheme that could automatically capture hand dual-view images and annotate the hand attitude angle values. In the second stage, we trained the CNN feature extractor separately on the acquired dual-view hand images to extract the dual-view deep features applicable to hand attitude regression and serially stitch the features of two different views. Finally, the ensemble learning algorithm was used to regress the hand attitude angle, which fit the hand features and the three-axis attitude angles of the hand, to establish the mapping between the hand image and the hand attitude angle. During the model test phase, we designed and trained the hand detection network. It could perform real-time hand detection on the images captured by the dual-view camera, to reduce the error in the application of real-time hand attitude estimation.

## 4. Methodology

### 4.1. Data Acquisition

**Figure 2:**
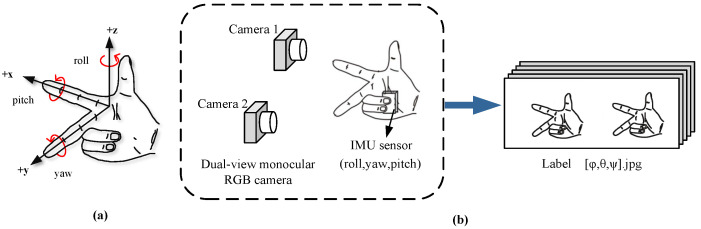
(**a**) Cartesian coordinate hand posture. (**b**) Diagram of dual-view data acquisition.

The problem to be solved in this work was the 3D hand attitude estimation for a fixed hand posture. For example, we first determined the predicted Cartesian coordinate system’s hand posture, as shown in Figure 2a, which could be regarded as a rigid object. Based on this assumption, the hand was restricted to three degrees of freedom in a pose. Its posture in three-dimensional space could be represented by the three-axis attitude angles, which consisted of the pitch angle θ, yaw angle ψ, and roll angle φ. Based on these angle values, we could recover the hand pose in 3D space. High-precision and high-quality training datasets are determinative for the performance of learning-based models. However, due to the deep blurring of RGB images, it is difficult to annotate them accurately, and the currently available datasets cannot satisfy our requirements for simultaneous acquisition of dual-view hand images and fixed hand postures. Therefore, we propose a fast construction method for 3D hand posture datasets based on dual-view cameras and an IMU sensor, which can quickly capture dual-view 2D hand image sets and automatically attach the corresponding three-axis attitude angle labels. The angle representing the hand posture comes from the coordinate system of the IMU sensor that collects posture data. In the beginning, we set the coordinate system of the IMU sensor to be the same as the world coordinate system. The operation is shown in Figure 2b, the IMU sensor is first placed in the palm, and the predicted hand posture is maintained. Then, we turn the wrist at a constant speed to rotate the hand around the 3D spatial coordinate axis. We use a dual-view camera to capture RGB images from two different views of the hand during rotation at regular intervals, and we use the dual-view camera to capture the hand images from two different views of the hand at regular intervals. While taking pictures, the three-axis attitude angle values output by the IMU sensor are recorded. Since we do not use the binocular stereo vision principle, the requirements for the placement position and viewing angle of the dual-view camera in this method are not so strict. We require that the dual-view camera is more capable of capturing a complete hand image, and the camera position when testing should be the same as the camera position when capturing images in the training set. Due to the different distances between the two cameras, the visual blind area is different. The greater the camera distance, the greater the blind area. This paper used a head-mounted dual-view camera. Therefore, we suggest that the baseline distance of the two cameras should be close to the distance between human eyes, and the minimum baseline should not be less than the distance between human eyes. This method can be programmed to automatically acquire, annotate, and save data, with the human hand only needing to rotate within the specified space angle. Therefore, the method is easy to operate, has low hardware equipment requirements, and has high data accuracy.

### 4.2. Hand Detection

In performing real-time hand attitude estimation, the camera may capture images that do not contain hands due to various factors. The attitude estimation of these images without hands not only affects our prediction results but also increases the computational cost of real-time estimation. Especially in the application of hand-posture-based human–computer interaction, the wrong attitude data output may have a greater impact on the system. Therefore, before estimating the hand attitude, we trained a hand detection model to reject invalid images.

The YOLO family of algorithms is a magnificent work in the field of target detection. YOLOv3 [32] has made further improvements based on the previous version. It uses Darknet-53 as the basic network and also uses a multiscale prediction approach. Through the above adjustments and improvements, the detection speed and accuracy of YOLOv3 are balanced, and the problem of inaccurate detection of small objects is solved. In the prediction phase of attitude estimation, we first trained the YOLOv3-based hand detection model to detect human hands on the images captured by the camera, which greatly improved the efficiency and accuracy of our real-time prediction.

### 4.3. Hand Attitude Estimation of Monocular RGB Images

The self-occlusion characteristics of the human hand in monocular RGB images largely affect the training and prediction of the hand attitude estimation model. This problem can be weakened by using dual-view images. The multiview hand images captured by cameras with different perspectives have certain complementarity in the description of hands. Training hand attitude estimation models for each monocular view image separately and then fusing the prediction results of the two models can improve the performance of the model to a certain extent.

**Figure 3:**
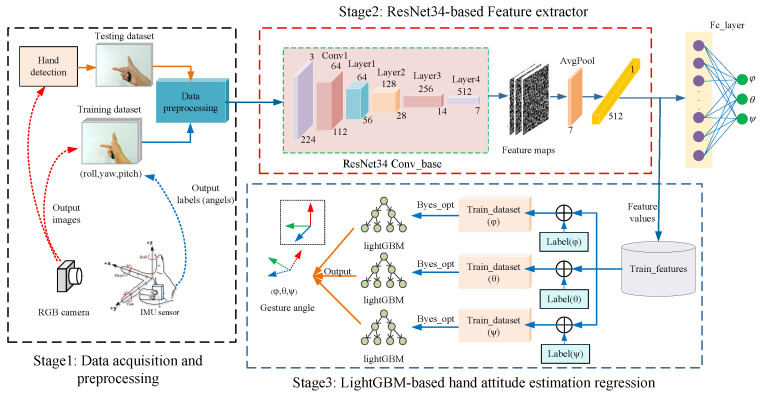
Hand attitude estimation process for each monocular view.

For the monocular hand images captured at the same time from different views, we designed hand attitude estimation models and finally fused them. Figure 3 shows the method of training the hand attitude estimation model for monocular view images. For the training of the model, the idea we adopted was to train the CNN feature extractors of each monocular view image and use them to extract the deep features of different view images. We performed serial stitching on the features extracted from the monocular images of different views obtained at the same time and then used the regression algorithm of the ensemble learning to replace the full connection layer of the CNN to regress the hand’s three-axis attitude. In the prediction, we first used the hand detection model to detect the hand in the captured image and then estimated the three-axis attitude of the image containing the hand.

This dual-view-based hand attitude estimation method can also be considered an ensemble learning idea, which integrates the results of different monocular view models. It can effectively weaken the problem of hand self-occlusion and solve the problem of randomness in the decision-making of a single model. Next, we present the feature extractor and hand attitude regressor designed in this paper.

### 4.4. Design of Feature Extractor

Compared with traditional methods of manually extracting image features, deep learning can automatically extract deep features. It does not need to design the feature extraction detection operator manually and solves the cumbersome feature engineering problem in machine learning. Moreover, the features extracted based on deep learning are all trained by the network and are closely related to the training samples and labels.

In this work, a convolutional neural network (CNN) was used for the feature extraction of this hand attitude estimation. A CNN is a deep neural network with a convolutional structure, which mainly consists of convolution layers, pooling layers, activation functions, and fully connected layers. Convolutional neural networks effectively reduce the number of parameters in the network by using local connectivity, weight sharing, and pooling operations to speed up network training, reduce computational resource consumption and alleviate the overfitting problem of the model.

The process of feature extraction by a CNN is the process of constantly learning the filter parameters in the convolution layer. Different features can be extracted by using different filters. The filters are the weights that perform the convolution operation. We wanted to train the network to extract a set of features that were suitable for our task by finding an optimal set of filter parameters.

**Figure 4:**
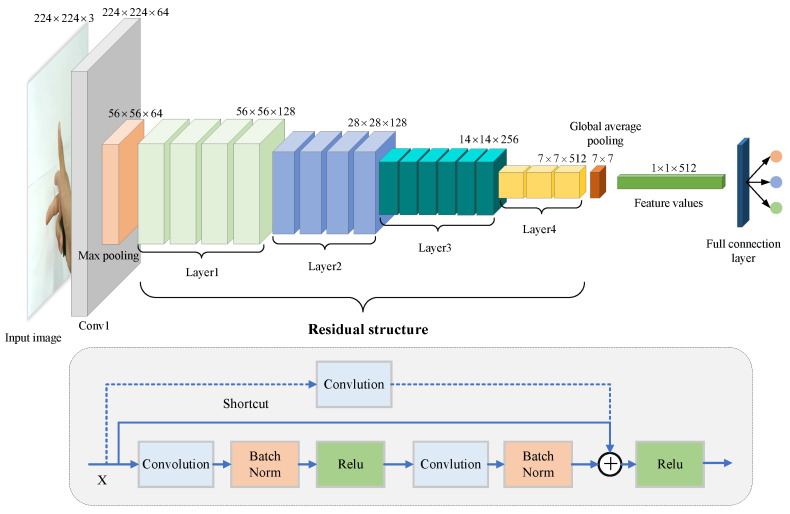
The ResNet34 structure diagram.

Theoretically, the deeper the convolutional neural network, the more abstract and richer the features it extracts. However, a simple layer stacking of the network leads to the disappearance or explosion of the gradient and the degradation of the model. For the former, we can overcome it by standardizing the data, initializing the network weight, and using the batch normalization layer. To address the problem of model degradation, in 2015, KaiMing He et al. proposed ResNet [33]. As shown in Figure 4, ResNet uses the residual structure, which reduces the network training time and makes it easier for the network to converge. It also uses the batch normalization (BN) layer, which can overcome the gradient disappearance and explosion problems. As a result, ResNet can build ultradeep network structures, often used as extraction networks for deep features.

Existing studies often use pretrained weights from ResNet directly for feature extraction. Considering the relatively homogeneous content of our dataset, extracting deep features using pretrained weights alone could not meet the needs of the hand attitude regression task in this study. Therefore, we further trained ResNet34 with the hand attitude angle as the label to fit the hand image and hand attitude angle data, and the convolutional basis part of the model after training could then be used as a feature extraction network.

**Figure 5:**
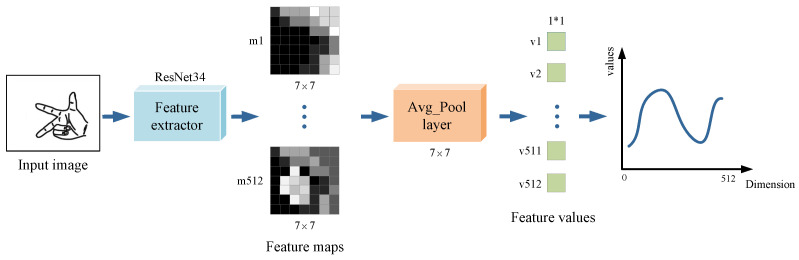
Hand image feature extraction based on ResNet34.

We used this method to train feature extractors $Fe_{1}$ and $Fe_{2}$ for captured dual-view monocular hand images. As shown in Figure 5, for the monocular image of each view, we extracted the 512-dimensional deep features of size 1×1 before the fully connected layer after it was input to ResNet34. For the dual-view images captured at the same moment, after extracting the dual-view features, respectively, they were serially stitched into the 1024-dimensional combined feature data $Fv_{1∥2}$.

### 4.5. Design of Hand Attitude Regressor

After extracting the deep features of the dual-view hand image, we used these feature data with the original image’s corresponding hand attitude angle labels to construct a new feature regression dataset and then trained the hand attitude regression model. Due to the high dimensionality of combined feature data, the hand attitude regression using only a single model is susceptible to random factors. Therefore, we adopted an ensemble learning algorithm with a stronger fitting ability to replace the CNN’s fully connected layer as the top regressor of the model and achieve the regression from hand image feature data to three-axis attitude data. By establishing the mapping between the hand image feature set and the three-axis attitude set, we obtained the mapping between the original image set and the three-axis attitude set. At the same time, we improved the performance of the single CNN with the idea of ensemble learning.

Among the various regression algorithms for ensemble learning, LightGBM, a light gradient boosting machine proposed by Microsoft in 2017, is another evolutionary version of the GBDT model. It continues the ensemble learning approach of XGBoost, as shown in Equation (1), and its objective function similarly adds regularization $\Omega (f_{j})$, a measure of model complexity, to the loss of computing the true value $y_{i}$ of the sample and the predicted value ${\hat{y}}_{i}^{\left(t\right)}$, effectively preventing the overfitting of the model.

(1) $$\mathrm{argmin}Obj^{\left(t\right)}=\sum_{i=1}^{N}L\left(y_{i},{\hat{y}}_{i}^{\left(t\right)}\right)+\sum_{j=1}^{t}\Omega \left(f_{j}\right)$$

LightGBM has been optimized and improved for finding the optimal segmentation point by using a data structure built on the histogram algorithm instead of the presorted algorithm, sacrificing some accuracy in exchange for training speed and saving memory space consumption. It proposes the gradient-based one-sided sampling (GOSS) algorithm for reducing the number of training samples and the mutually exclusive feature bundling (EFB) for reducing the number of training features. It solves the problem that the histogram algorithm has worse processing time complexity than the presorted algorithm for sparse data while improving the training speed.

**Figure 6:**
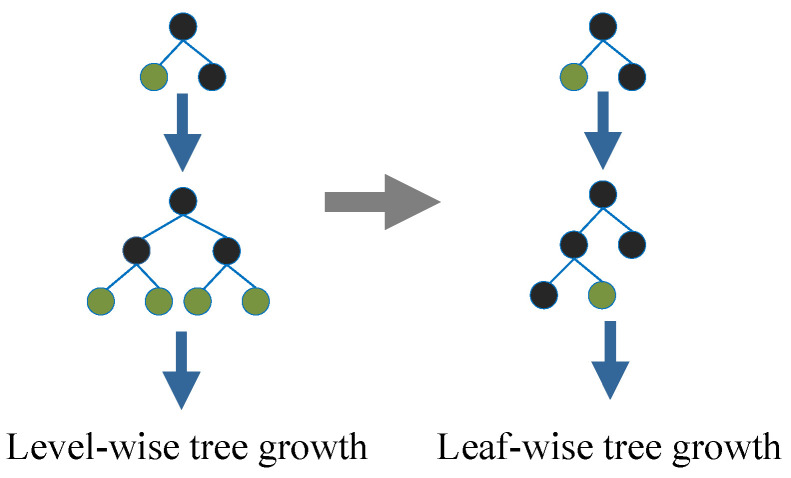
Levelwise and leafwise tree growth.

As shown in Figure 6, in terms of tree growth strategy, LightGBM differs from XGBoost’s levelwise tree growth strategy and proposes a more efficient leafwise growth strategy, which can reduce errors and obtain a better accuracy. Therefore, the algorithm has fast training speed and low memory occupation characteristics compared with XGBoost.

We used LightGBM to construct a hand attitude regressor on the feature regression dataset, fitting the hand image feature set and the attitude angle data, whose input was the features of our extracted and stitched-together dual-view images, and whose output was the hand attitude angle corresponding to the features. There are many hyperparameters for hand attitude regression using LightGBM. Grid search and random search are the usual means of hyperparameter tuning, but they are inefficient when the complexity of the model is high and the number of hyperparameters is large.

Snoek [34] first applied Bayesian optimization methods to the selection of hyperparameters for machine learning algorithms and achieved good tuning results in many machine-learning-related studies [35,36]. We adopted Bayesian optimization for the hyperparametric tuning of the LightGBM attitude regression model. Bayesian optimization is the process of fitting the true distribution of f(x) through a probabilistic surrogate model based on presampled observations *x* without knowing the true objective function f(x), and adding additional sampling points through the acquisition function. Then, reacquiring the posterior probability distribution based on the set of sampled observations and continuously iteratively updating it to eventually find the optimal point and optimal value of the objective function. Compared to grid search and random search, it makes full use of the prior knowledge of previous observations at each iteration for the next step of optimization, speeding up the optimization search process of the model. The optimization expression of the model is shown in Equation (2). The set of observation points *x* is the hyperparameter space of LightGBM, and the objective function f(x) to be optimized is the root-mean-square error (RMSE) or mean absolute error (MAE) of the fit of LightGBM to the features of the hand image and the hand attitude data, and the parameter at the minimum of the error is the optimal hyperparameter $x^{*}$.

(2) $$x^{*}=\underset{x\in \chi }{\mathrm{argmin}}f\left(x\right)$$

For the probabilistic surrogate model, we used the tree-structured Parzen estimators (TPE) process, where the acquisition function used the exponential integral (EI) function as in Equation (3), and its next observation was chosen based on maximizing the expectation of the improvement lift.

(3) $$EI\left(x\right)=E\left[\max\left\{0,f\left(x^{*}\right)-f\left(x\right)\right\}\right]$$

In summary, the main steps of the hand attitude estimation method in this paper were to first extract features from the image and to use the regression algorithm in ensemble learning to achieve the regression of the hand attitude for the extracted features. The hand attitude estimation method could be divided into two parts: training and prediction of the model. First, we used the hold-out method to divide the dataset according to the scale and trained the feature extractors ${Fe}_{1}$ and ${Fe}_{2}$ separately for the training set’s sample data of each view. The extracted hand features of two different views were serially spliced to obtain the combined feature $F_{{train}_{1∥2}}$. We then trained a LightGBM attitude regressor based on a Bayesian optimization algorithm on the combined features. In the prediction, we first detected the hand and then used ${Fe}_{1}$ and ${Fe}_{2}$ to extract the features of the dual-view image containing the hand. After stitching the dual-view features, we used the LightGBM attitude regressor to predict the three-axis attitude of the hand.

## 5. Experimental Design and Results

To verify the effectiveness of the hand attitude estimation algorithm proposed in this paper, experiments were designed and tested on a PC with an Intel Core i10, 16 GB of RAM, and a RTX2060 GPU.

### 5.1. Datasets

**Figure 7:**
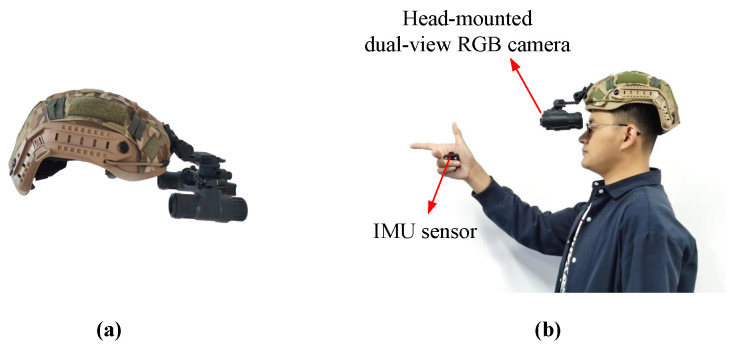
(**a**) Head-mounted dual-view RGB camera. (**b**) Schematic diagram of data acquisition.

**Figure 8:**
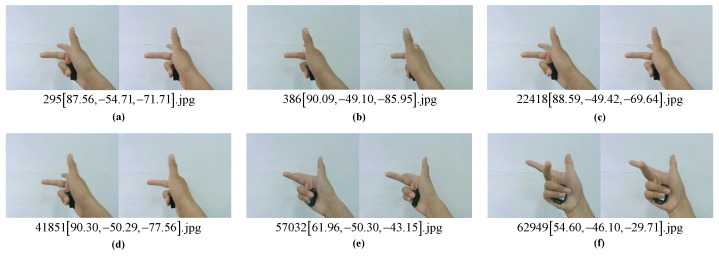
A partial sample of C-Posehand dataset. (**a**–**f**) are six samples in the dataset.

We collected and constructed a dual-view hand attitude estimation dataset (C-Posehand dataset) with a right-handed Cartesian coordinate system hand as the target hand type following the data collection method described in Section 4.1. As shown in Figure 7, we used a head-mounted dual-view camera to capture RGB images of different views of the hand and placed an IMU sensor with a Bluetooth function in the palm to collect the three-axis attitude angles of the hand. The hand images and attitude angles were automatically captured, annotated, and saved using Python programming. Using this method, we only needed to maintain the predicted hand type and make the hand rotate slowly at a constant speed within the spatial angle range where the wrist could rotate. As shown in Figure 8, a partial sample of our dataset, we obtained 70,000 sets of dual-view RGB hand images and the corresponding three-axis attitude data.

### 5.2. Hand Detecting Experimental Setup

**Figure 9:**
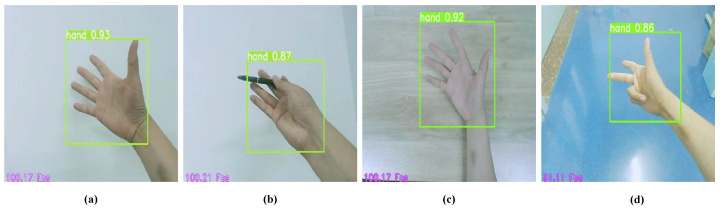
Sample test of the YOLOv3 hand detection model. (**a**–**d**) show the test effect of different gestures and different backgrounds.

We selected the hand detection dataset proposed by S. Narasimhaswamy et al. in [37] to train the YOLOv3 hand detection model, which consists of images and labels. Images include images from the TV-Hand dataset and images from the COCO-Hand dataset, with a total of 32,410 color frames. Labels represent the category to which the target belongs, the x and y coordinates of the label frame, and the width and height of the label frame. We used this dataset to train the hand detection network based on YOLOv3. Figure 9 shows a test sample of our trained hand detection model with good recognition accuracy and Fps values.

### 5.3. Metrics

The three-axis attitude angles (φ,θ,ψ) were regressed in our suggested hand attitude estimation method. In order to evaluate the respective regression predictions of the model for the roll angle φ, pitch angle θ, and yaw angle ψ for the test samples, which are provided in Equations (4) and (5), we used the mean squared error (MSE) and the mean absolute error (MAE).

(4) $$MSE_{\alpha }=\frac{1}{n}\sum_{i=1}^{n}{\left(\alpha_{i}-{\hat{\alpha }}_{i}\right)}^{2}$$

(5) $$MAE_{\alpha }=\frac{1}{n}\sum_{i=1}^{n}\left|\alpha_{i}-{\hat{\alpha }}_{i}\right|$$

where *n* is the number of samples and α represents one of the three-axis attitude angles.

We evaluated the overall effects of the model on the three angles of a single sample using the root-mean-square error (RMSE) and mean absolute error (MAE), which are expressed in Equations (6)–(8).

(6) $$MSE_{i}=\frac{1}{3}\left({\left(\varphi_{i}-{\hat{\varphi }}_{i}\right)}^{2}+{\left(\theta_{i}-{\hat{\theta }}_{i}\right)}^{2}+{\left(\psi_{i}-{\hat{\psi }}_{i}\right)}^{2}\right),i=1,2,\ldots ,n$$

(7) $$RMSE_{i}={\left(MSE_{i}\right)}^{1/2},i=1,2,\ldots ,n$$

(8) $$MAE_{i}=\frac{1}{3}\left(|\varphi_{i}-{\hat{\varphi }}_{i}\left|+\right|\theta_{i}-{\hat{\theta }}_{i}\left|+\right|\psi_{i}-{\hat{\psi }}_{i}|\right),i=1,2,\ldots ,n$$

We also provided the evaluation metric for each sample’s prediction accuracy. We considered the model’s prediction for this sample to be accurate when the MAE and RMSE score predicted from the three angles of a single sample were within the designated allowable range. The accuracy rate discussed in this work was defined as the ratio of the number of samples in the statistical test set that met the error threshold to the total number of samples in the test set. The formulas for it are (9) and (10).

(9) $$\mathrm{Accuracy}=\frac{1}{n}\sum_{i=1}^{n}I\left(RMSE_{i}<b\right)\times 100\%$$

(10) $${\mathrm{Accuracy}}^{\prime }=\frac{1}{n}\sum_{i=1}^{n}I\left(MAE_{i}<b\right)\times 100\%$$

In addition, the real-time fluency of the model prediction is also a vital evaluation metric; we used Fps (frames per second) to evaluate the speed and fluency of the real-time model prediction. The formula is as follows (11).

(11) $$Fps=\frac{\;\mathrm{Num}\;(\;\mathrm{frame}\;)}{\;\mathrm{Time}\;(\;\mathrm{elapsed}\;)}$$

### 5.4. Feature Extracting Experimental Setup

We trained feature extractors ${Fe}_{1}$ and ${Fe}_{2}$ suitable for hand attitude regression using ResNet34 for two different views of the images in the dataset, respectively. For the data collected under each view, we changed the classifier output dimension of the top layer of ResNet34 to the same three dimensions as our three-axis attitude, so that the network directly output the three-axis attitude angles of the hand.

Before training the model, we divided the dataset into a training set, a validation set, and a test set using the hold-out method with a 7:2:1 ratio of stratified sampling. The hand images in the training set and the three-axis attitude angles labeled with the image names were read, and the read data were subjected to preprocessing operations including changing the image size, conversion to a tensor format, and normalization operations. It was then fed to the ResNet34 in batches in the form of a minibatch for training, using the method of pretrained weights in transfer learning to speed up network convergence. The training learning rate was set to 0.001 and the number of iterations was 40, with the training ending after saving the weights of the training model with the highest accuracy.

**Figure 10:**
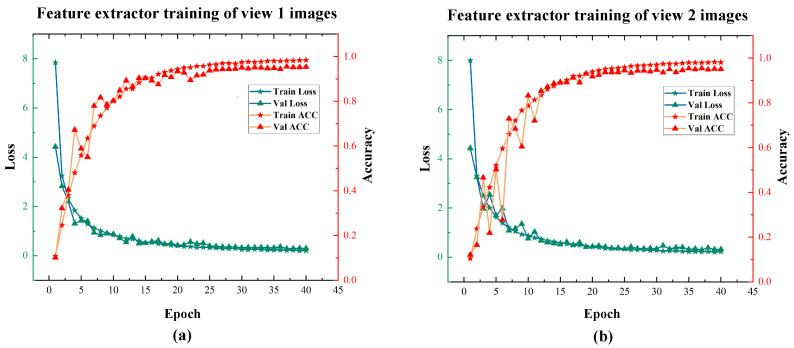
The training process curve of feature extractor based on ResNet34. (**a**) represents view 1, (**b**) is view 2.

The RMSE of a single sample was used as the evaluation metric during training, and a sample was considered to be correctly predicted when the RMSE of a single sample was less than 2°. The convergence of the smooth L1 loss and the accuracy curve of the model on the training and validation sets during the training process are shown in Figure 10.

**Figure 11:**
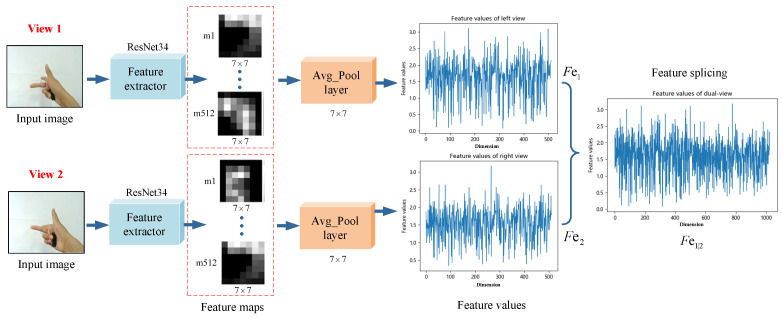
Extracting depth features from dual-view hand images using ResNet34.

As seen from the training curves, our model reached convergence and achieved good prediction results on the validation set. The trained model could already extract the depth features of hand attitude regression well. We used the convolutional structure part of the trained ResNet to perform the feature extraction on the dual-view hand images of the C-PoseHand dataset, outputting two sets of 512-dimensional feature values before the fully connected layer. As shown in Figure 11, for the dual-view image captured simultaneously, we extracted the depth features using the feature extractors of their respective views separately, then serially stitched the two sets of 512-dimensional into a set of 1024-dimensional combined feature data.

### 5.5. LightGBM Hand Attitude Regression

The fully connected layer in ResNet34 was replaced with LightGBM as the network’s top predictor, and regression was used to fit the features taken from the C-PoseHand dataset and the related hand attitude angles. We built three LightGBM models to fit regressions to each of the three-dimensional hand attitude data because our hand attitude data were three-dimensional.

**Figure 12:**
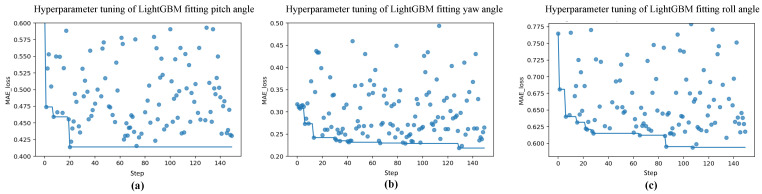
Variation of L1 loss of the validation set during the optimization iterations of LightGBM. (**a**–**c**) indicate the tuning process when LightGBM fits the pitch, yaw, and roll angles, respectively. The blue circles in the figure indicate the L1 loss values of the model on the validation set for each iteration.

In order to find the best set of hyperparameters to train and save the LightGBM hand attitude estimation model, we used the Hyperopt package [38] for the Bayesian optimization and tuning of the parameters during the training of the LightGBM hand attitude regression model. Using Bayesian optimization for hyperparameter tuning, Figure 12 shows the iterative decline in the MAE Loss on the validation set for the model trained with various parameter combinations.

**Figure 13:**
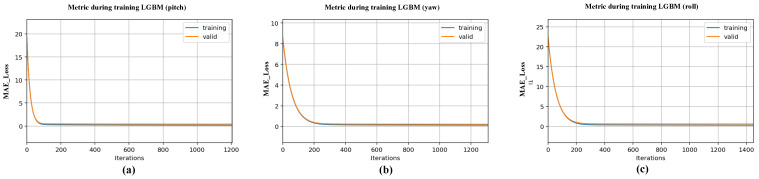
Loss changes in LightGBM training process. (**a**–**c**) show the training process when LightGBM fits pitch, yaw, and roll angles, respectively.

The optimal hyperparameters for the LightGBM hand pose regression model after Bayesian optimization and tuning are summarized in Table 1. The parameter *num_iterations*, which represents the number of model iterations, was determined dynamically using an early stop mechanism during model training. The number of iterations was set to *best_iteration* when the loss value on the validation set no longer decreased after *I* iterations, and in this experiment, the *I* was taken to be 20. The LightGBM model for hand pose estimation (ConvLGBM 2) was trained using Bayesian tuning of the optimal hyperparameters. Figure 13 shows the loss curve of the LightGBM hand attitude estimation model during training. Table 2 gives the MSE, MAE, and $R^{2}$ metrics and the training time of the model on the training and test sets after the model was trained.

### 5.6. Performance Comparison

For comparative trials, we make reference to relevant algorithms. First, we trained the hand attitude estimation model (ConvLGBM 1) of monocular images using one of the view images from our dataset as an example and the hand attitude estimation approach described in this paper. Similarly, while using the LightGBM attitude regression to the characteristics of monocular images, we adjusted the hyperparameters based on a Bayesian optimization. Table 3 displays the ideal values, while Table 4 displays the outcomes of model training using these parameters.

**Figure 14:**
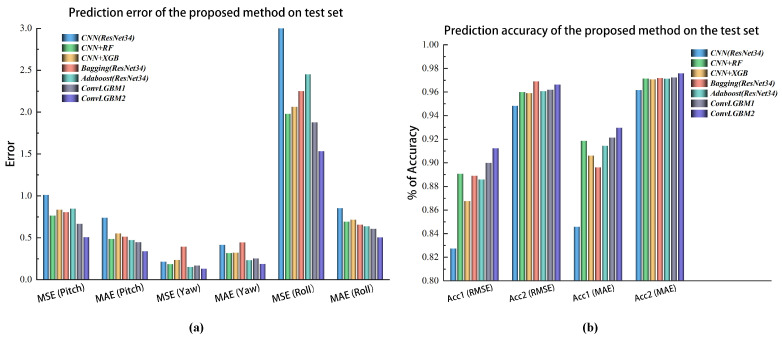
Comparison of test metrics on the C-PoseHand dataset between the proposed method and other methods. (**a**) Comparison of the prediction error. (**b**) Comparison of the prediction accuracy.

In addition, this paper proposed an ensemble learning method for a CNN, so we used the feature extraction network ResNet34 trained in this paper as a single convolutional neural network model for comparison with this method, which finally enabled hand attitude regression through a fully connected layer. We also referred to existing related research methods using ensemble learning methods for image processing and adapted them to the hand dataset presented in this paper. We referred to [28] and experimented with XGBoost regression on the features extracted from a CNN (CNN+XGBoost), whose main parameter settings were “*subsample*”: 1, “*max_depth*”: 4, “*eta*”: 0.15, “*gamma*”: 0, “*lambda*”: 1, and “*alpha*”: 0.2. Furthermore, we did experimental tests (CNN+RF) using a representative bagging algorithm, random forest, whose main parameter settings were “*max_depth*”: 22.0, “*max_features*”: 19.0, “*min_impurity_decrease*”: 0.0, “*n_estimators*”: 98.0. We referenced [24,39] and fused five different ResNet34 hand attitude estimation models for our dataset using the bagging algorithm with ResNet34 as the base learner, and the prediction output of their fusion models was the mean of the predictions from the five base learners. ResNet34 was also boosted using the AdaBoost algorithm, we trained five different ResNet34s with the same update iterations, and the fusion output was also the mean of the predictions of the base learners. We have collated the MSE and MAE metrics predicted by the different algorithms in Table 5 and present them as histograms in Figure 14a.

As can be seen from Table 5 and Figure 14a, the 3D hand attitude prediction method proposed in this paper predicted smaller MSE and MAE metrics using a single view than some other methods, and in particular, the ConvLGBM 2 model trained using images from a dual view had a significant performance improvement compared to other algorithms. To evaluate the overall effect of the model on the three-axis attitude prediction of a single sample, we also compared the proportion of samples within 1° (Acc1) and 2° (Acc2) for RMSE and MAE for a single sample for each model, and the test results are shown in Table 6 and Figure 14b. The comparison results show that the proposed method in this paper had a higher prediction accuracy than the other related methods and had a significant improvement over the prediction accuracy using a single CNN, especially for the model prediction error of less than 1°. It can be seen that the proposed hand attitude estimation method based on dual-view hand images had a better prediction effect than that using monocular view images, which shows that it could effectively weaken the impact of hand self-occlusion on attitude estimation.

To verify the efficiency of the hand attitude estimation method described in this paper when performing real-time prediction, we tested the Fps metrics of this method and other methods for real-time prediction, and the test results are shown in Table 7.

## 6. Conclusions

In this study, we offered a method for estimating the three-axis attitude angles of a fixed hand posture in three dimensions using dual-view RGB cameras, combining the advantages of deep learning and ensemble learning. First, using dual-view cameras and an IMU sensor, we suggested a quick technique for building 3D hand attitude datasets and created the C-PoseHand dataset for estimating hand attitude. Then, we trained CNN-based image feature extractors to extract the dual-view images’ features and analyze them serially. Finally, we implemented attitude regression for the stitched features using LightGBM, which was based on Bayesian optimization. A YOLOv3-based hand identification model was trained to filter out unreliable images before the hand attitude was estimated. The results of the experiments demonstrated that the proposed approach might successfully resolve the hand self-occlusion issue and achieved 3D hand attitude estimate with only two normal RGB cameras.

In the fields of augmented reality, virtual reality, and human–computer interaction, this hand attitude estimation approach can be used. We want to employ this hand attitude estimation method in situations where the controlled item does not need a lot of degrees of freedom, which is the focus and direction of our upcoming research. This approach, in our opinion, is adaptable, simple to use, and has light hardware requirements. Different hand postures can be set according to the application requirements using the method presented in this study for data acquisition and training hand attitude estimation models. However, as our method uses a dual model trained by dual-view images, the efficiency of real-time prediction is reduced, which we will optimize and improve in a later study.

## Figures and Tables

**Table 1:** Bayesian hyperparameters optimization results for dual-view images.

Hyperparameters	Best_Params		
	LGBM (θ)	LGBM (ψ)	LGBM (φ)
num_leaves	94	195	202
learning_rate	0.16121	0.02335	0.05533
max_depth	10	9	9
min_data_in_leaf	9	6	13
min_gain_to_split	0.01718	0.03966	0.10516
feature_fraction	0.91663	0.78078	0.81469
bagging_fraction	0.98435	0.78186	0.99526
bagging_freq	3	2	7

**Table 2:** Training results of LightGBM model for dual-view images.

Scores	LGBM (θ)	LGBM (ψ)	LGBM (φ)
Best iteration	1309	2825	768
Training time (s)	65.7854	155.1422	31.5946
Train L1	0.12444	0.10903	0.23567
Valid L1	0.34008	0.19558	0.51301
Valid MSE	0.51491	0.13813	1.5398
Valid $R^{2}$	0.9991	0.99886	0.99793

**Table 3:** Bayesian hyperparameters optimization results for single-view images.

Hyperparameters	Best_Params		
	LGBM (θ)	LGBM (ψ)	LGBM (φ)
num_leaves	228	243	107
learning_rate	0.14475	0.07746	0.21091
max_depth	10	8	9
min_data_in_leaf	19	14	26
min_gain_to_split	0.00124	0.00948	0.00845
feature_fraction	0.75504	0.79632	0.87757
bagging_fraction	0.97485	0.91240	0.99871
bagging_freq	6	1	5

**Table 4:** Training results of LightGBM model for single-view images.

Scores	LGBM (θ)	LGBM (ψ)	LGBM (φ)
Best iteration	1305	2235	1073
Training time (s)	29.1329	38.4382	21.3928
Train L1	0.12698	0.09591	0.16236
Valid L1	0.35877	0.20576	0.51776
Valid MSE	0.62873	0.15642	1.9347
Valid $R^{2}$	0.9989	0.9987	0.9974

**Table 5:** Comparison of the proposed method to other methods.

Methods	MSE			MAE		
	MSE (θ)	MSE (ψ)	MSE (φ)	MAE (θ)	MAE (ψ)	MAE (φ)
CNN(ResNet34)	1.0182	0.2217	3.0249	0.7455	0.4226	0.8625
CNN+RF	0.7715	0.1934	1.9853	0.4936	0.3252	0.6988
CNN+XGB	0.8424	0.2432	2.0697	0.5604	0.3282	0.7239
Bagging(ResNet34)	0.8133	0.3997	2.2565	0.5201	0.4541	0.6645
Adaboost(ResNet34)	0.8545	0.1576	2.4586	0.4814	0.2385	0.6438
ConvLGBM 1(Ours)	0.6741	0.1771	1.8834	0.4555	0.2622	0.6151
ConvLGBM 2(Ours)	0.5149	0.1381	1.5398	0.3448	0.1955	0.5134

**Table 6:** Comparison of accuracy between the proposed method and other methods.

Methods	RMSE		MAE	
	Acc1	Acc2	Acc1	Acc2
CNN(ResNet34)	82.78%	94.88%	84.63%	96.21%
CNN+RF	89.12%	96.04%	91.91%	97.19%
CNN+XGB	86.80%	95.96%	90.65%	97.12%
Bagging(ResNet34)	88.95%	96.95%	89.66%	97.23%
Adaboost(ResNet34)	88.63%	96.12%	91.50%	97.18%
ConvLGBM 1(Ours)	90.04%	96.24%	92.19%	97.29%
ConvLGBM 2(Ours)	91.28%	96.68%	93.02%	97.63%

**Table 7:** Comparison of Fps metrics for real-time prediction by different methods.

Methods	Fps
CNN(ResNet34)	116.5081
CNN+RF	91.2437
CNN+XGB	91.8226
Bagging(ResNet34)	70.3915
Adaboost(ResNet34)	64.5963
ConvLGBM 1(Ours)	92.4828
ConvLGBM 2(Ours)	86.5126

## Data Availability

The data presented in this study are available on request from the corresponding authors.
